# Supplementary material for: Chemistry and Bioactivity of Microsorum scolopendria (Polypodiaceae): Antioxidant Effects on an Epithelial Damage Model
Source: Molecules. 2022 Aug 25;27(17):5467. doi: 10.3390/molecules27175467 (PMC9457714; doi:10.3390/molecules27175467)
Supplement: Supplementary file 1 [file molecules-27-05467-s001.zip › molecules-1868076-supplementary.pdf]

Supplementary Material

Table S1. Phenolic acids found in *MS* by RP-HPLC-MS/MS in negative mode.

					HAE				RAE			
Name	Molecular Formula	Subclass	Expected Mass (m/z)	Expected Retention Time (s)	Retention Time (s)	Retention Time Variation (s)	Observed Mass (m/z)	Mass Variation (m/z)	Retention Time (s)	Retention Time Variation (s)	Observed Mass (m/z)	Mass Variation (m/z)
24-methyl cholesterol ferulate	$C_{38}H_{56}O_4$	Hydroxycinnamic acid	576,85	0,77	NA	NA	NA	NA	0,76	0,01	578,87	-2,02
Avenanthramide 2F	$C_{17}H_{15}NO_6$	Hydroxycinnamic acid	329,3	18,42	NA	NA	NA	NA	18,65	-0,23	329,3	0
4-glycogallic acid	$C_{13}H_{16}O_{10}$	Hydroxybenzoic acid	332,26	20,92	20,91	0,01	332,25	0,01	20,93	-0,01	332,25	0,01
Galloyl glucosa	$C_{13}H_{16}O_{10}$	Hydroxybenzoic acid	332,07	20,92	20,91	0,01	332,25	-0,18	20,93	-0,01	332,25	-0,18
Sinapine	$C_{16}H_{24}NO_5$	Hydroxycinnamic acid	310,37	21,8	21,62	0,18	310,36	0,01	NA	NA	NA	NA
Ellagic acid glucoside	$C_{20}H_{16}O_{13}$	Hydroxybenzoic acid	464,34	22,52	22,51	0,01	464,32	0,02	22,53	-0,01	464,32	0,02
Protocatechuic acid 4-O-glucoside	$C_{13}H_{16}O_9$	Hydroxybenzoic acid	316,26	22,29	22,94	-0,65	316,26	0	22,97	-0,68	316,26	0
P-coumaroyl tartaric acid	$C_{13}H_{12}O_8$	Hydroxycinnamic acid	296,23	23,55	23,57	-0,02	296,23	0	23,6	-0,05	296,23	0
Caffeoyl tartaric acid	$C_{13}H_{12}O_9$	Hydroxycinnamic acid	312,23	23,85	23,86	-0,01	312,23	0	23,88	-0,03	312,23	0
4-hydroxybenzoic acid 4-O-glucoside	$C_{13}H_{16}O_8$	Hydroxybenzoic acid	300,26	25,42	25,44	-0,02	300,26	0	25,46	-0,04	300,26	0
Ellagic acid acetyl arabinoside	$C_{21}H_{16}O_{13}$	Hydroxybenzoic acid	476,34	25,59	25,57	0,02	476,35	-0,01	25,61	-0,02	476,35	-0,01
Ellagic acid acetyl-xyloside	$C_{21}H_{16}O_{13}$	Hydroxybenzoic acid	476,34	25,59	25,57	0,02	476,35	-0,01	25,61	-0,02	476,35	-0,01
Feruloyl tartaric acid	$C_{14}H_{14}O_9$	Hydroxycinnamic acid	326,26	28,7	28,7	0	326,27	-0,01	NA	NA	NA	NA

NA: Not among the most abundant in the extract.

Table S2. Phenolic acids found in *MS* by RP-HPLC-MS/MS in positive mode.

					HAE				RAE			
Name	Molecular Formula	Subclass	Expected Mass (m/z)	Expected Retention Time (s)	Retention Time (s)	Retention Time Variation (s)	Observed Mass (m/z)	Mass Variation (m/z)	Retention Time (s)	Retention Time Variation (s)	Observed Mass (m/z)	Mass Variation (m/z)
Dihydrocaffeic acid	$C_9H_{10}O_4$	Hydroxyphenyl propanoic acid	182,17	18,69	18,83	-0,14	182,16	0,01	18,76	-0,07	182,16	0,01
Homovanillic acid	$C_9H_{10}O_4$	Hydroxyphenylacetic acid	182,17	18,69	18,83	-0,14	182,16	0,01	18,76	-0,07	182,16	0,01
Caffeic acid	$C_9H_8O_4$	Hydroxycinnamic acid	180,16	18,75	18,61	0,14	180,15	0,01	18,61	0,14	180,15	0,01
Gallic acid 4-O-glucoside	$C_{13}H_{16}O_{10}$	Hydroxybenzoic acid	332,26	20,92	20,92	0	332,27	-0,01	21	-0,08	332,25	0,01
Galloyl glucose	$C_{13}H_{16}O_{10}$	Hydroxybenzoic acid	332,26	20,92	20,92	0	332,27	-0,01	21	-0,08	332,25	0,01
Ellagic acid glucoside	$C_{20}H_{16}O_{13}$	Hydroxybenzoic acid	464,34	22,52	22,27	0,25	464,32	0,02	22,27	0,25	464,32	0,02
P-coumaroyl malic acid	$C_{13}H_{12}O_7$	Hydroxycinnamic acid	280,23	22,56	22,61	-0,05	280,23	0	22,59	-0,03	280,23	0
P-coumaric acid	$C_9H_8O_3$	Hydroxycinnamic acid	164,16	22,67	22,69	-0,02	164,15	0,01	22,85	-0,18	164,15	0,01
5-O-galloylquinic acid	$C_{14}H_{16}O_{10}$	Hydroxybenzoic acid	344,27	22,93	22,87	0,06	344,28	-0,01	23,1	-0,17	344,28	-0,01
Protocatechuic acid 4-O-glucoside	$C_{13}H_{16}O_9$	Hydroxybenzoic acid	316,26	22,93	23,08	-0,15	316,26	0	23,09	-0,16	316,26	0
P-coumaroyl tartaric acid	$C_{13}H_{12}O_8$	Hydroxycinnamic acid	296,23	23,55	23,63	-0,08	296,23	0	23,41	0,14	296,23	0
2-hydroxybenzoic acid	$C_7H_6O_3$	Hydroxybenzoic acid	138,12	23,56	23,47	0,09	138,13	-0,01	23,56	0	138,13	-0,01

3-hydroxybenzoic acid	$C_7H_6O_3$	Hydroxybenzoic acid	138,12	23,56	23,47	0,09	138,13	-0,01	23,56	0	138,13	-0,01
4-hydroxybenzoic acid	$C_7H_6O_3$	Hydroxybenzoic acid	138,12	23,56	23,47	0,09	138,13	-0,01	23,56	0	138,13	-0,01
Vanillic acid	$C_8H_8O_4$	Hydroxybenzoic acid	168,14	23,84	23,69	0,15	168,15	-0,01	23,66	0,18	168,15	-0,01
Caffeoyl tartaric acid	$C_{13}H_{12}O_9$	Hydroxycinnamic acid	312,23	23,85	23,64	0,21	312,22	0,01	24,08	-0,23	312,22	0,01
Sinapic acid	$C_{11}H_{12}O_5$	Hydroxycinnamic acid	224,21	23,91	24	-0,09	224,21	0	23,94	-0,03	224,21	0
Valoneic acid dilactone	$C_{21}H_{10}O_{13}$	Hydroxybenzoic acid	470,29	24,04	NA	NA	NA	NA	24,1	-0,06	470,3	-0,01
Gallic acid 3-O-gallate	$C_{14}H_{10}O_9$	Hydroxybenzoic acid	322,22	24,05	24,28	-0,23	322,22	0	23,93	0,12	322,22	0
4-hydroxybenzoic acid 4-O-glucoside	$C_{13}H_{16}O_8$	Hydroxybenzoic acid	300,26	25,42	25,45	-0,03	300,26	0	25,24	0,18	300,26	0
Ellagic acid acetyl arabinoside	$C_{21}H_{16}O_{13}$	Hydroxybenzoic acid	476,34	25,59	25,76	-0,17	476,35	-0,01	NA	NA	NA	NA
Ellagic acid acetyl-xyloside	$C_{21}H_{16}O_{13}$	Hydroxybenzoic acid	476,34	25,59	25,76	-0,17	476,35	-0,01	NA	NA	NA	NA
Caffeoyl aspartic acid	$C_{13}H_{13}NO_7$	Hydroxycinnamic acid	295,24	26,37	26,59	-0,22	295,25	-0,01	NA	NA	NA	NA
Hydrocaffeic acid	$C_9H_8O_5$	Hydroxycinnamic acid	196,16	26,42	26,62	-0,2	196,15	0,01	26,42	0	196,15	0,01
Gallic acid	$C_7H_6O_5$	Hydroxybenzoic acid	170,12	26,46	26,59	-0,13	170,12	0	26,69	-0,23	170,12	0
Ellagic acid	$C_{14}H_6O_8$	Hydroxybenzoic acid	302,19	26,55	26,64	-0,09	302,2	-0,01	26,3	0,25	302,2	-0,01
Avenanthramide 2P	$C_{16}H_{13}NO_5$	Hydroxycinnamic acid	299,28	26,61	26,67	-0,06	299,27	0,01	NA	NA	NA	NA
Caffeic acid 4-O-glucoside	$C_{15}H_{18}O_9$	Hydroxycinnamic acid	342,29	26,71	26,65	0,06	342,3	-0,01	26,78	-0,07	342,31	-0,02
Caffeoyl glucose	$C_{15}H_{18}O_9$	Hydroxycinnamic acid	342,29	26,71	26,65	0,06	342,3	-0,01	26,78	-0,07	342,31	-0,02
P-coumaric acid 4-O-glucoside	$C_{15}H_{18}O_8$	Hydroxycinnamic acid	326,29	27,32	27,27	0,05	326,31	-0,02	27,28	0,04	326,31	-0,02
P-coumaroylquinic acid	$C_{16}H_{18}O_8$	Hydroxycinnamic acid	338,31	27,73	27,88	-0,15	338,31	0	27,84	-0,11	338,31	0

2,3-dihydroxybenzoic acid	$C_7H_6O_4$	Hydroxybenzoic acid	154,12	35,26	35,25	0,01	154,12	0	35,3	-0,04	154,12	0
2,4-dihydroxybenzoic acid	$C_7H_6O_4$	Hydroxybenzoic acid	154,12	35,26	35,25	0,01	154,12	0	35,3	-0,04	154,12	0
2,5-dihydroxybenzoic acid	$C_7H_6O_4$	Hydroxybenzoic acid	154,12	35,26	35,25	0,01	154,12	0	35,3	-0,04	154,12	0
2,6-dihydroxybenzoic acid	$C_7H_6O_4$	Hydroxybenzoic acid	154,12	35,26	35,25	0,01	154,12	0	35,3	-0,04	154,12	0
Protocatechuic acid	$C_7H_6O_4$	Hydroxybenzoic acid	154,12	35,26	35,25	0,01	154,12	0	35,3	-0,04	154,12	0

NA: Not among the most abundant in the extract.

Table S3. Flavonoids found in *MS* by RP-HPLC-MS/MS in negative mode.

					HAE				RAE			
Name	Molecular Formula	Subclass	Expected Mass (m/z)	Expected Retention Time (s)	Retention Time (s)	Retention Time Variation (s)	Observed Mass (m/z)	Mass Variation (m/z)	Retention Time (s)	Retention Time Variation (s)	Observed Mass (m/z)	Mass Variation (m/z)
Kaempferol	$C_{15}H_{10}O_6$	Flavonol	286,23	20,99	21,01	-0,02	286,24	-0,01	21,01	-0,02	286,24	-0,01
Luteolin	$C_{15}H_{10}O_6$	Flavone	286,23	20,99	21,01	-0,02	286,24	-0,01	21,01	-0,02	286,24	-0,01
Scutellarein	$C_{15}H_{10}O_6$	Flavone	286,24	20,99	21,01	-0,02	286,24	0	21,01	-0,02	286,24	0
Cyanidin	$C_{15}H_{11}O_6$	Anthocyanin	287,24	21	21,02	-0,02	287,24	0	21	0	287,24	0
6-hydroxyluteolin	$C_{15}H_{10}O_7$	Flavone	302,23	21,13	21,14	-0,01	302,24	-0,01	21,15	-0,02	302,24	-0,01
Apigenin 7-O-glucuronide	$C_{21}H_{18}O_{11}$	Flavone	446,4	22,24	NA	NA	NA	NA	22,26	-0,02	446,36	0,04
Cirsimaritin	$C_{17}H_{14}O_6$	Flavone	314,29	22,64	NA	NA	NA	NA	22,63	0,01	314,29	0
Isorhamnetin	$C_{16}H_{12}O_7$	Flavonol	316,26	22,29	22,94	-0,65	316,26	0	22,97	-0,68	316,26	0
Nepetin	$C_{16}H_{12}O_7$	Flavone	316,26	22,29	22,94	-0,65	316,26	0	22,97	-0,68	316,26	0
Ramnetin	$C_{16}H_{12}O_7$	Flavonol	316,26	22,29	22,94	-0,65	316,26	0	22,97	-0,68	316,26	0
Daidzin	$C_{21}H_{20}O_9$	Isoflavonoid	416,38	24,32	24,3	0,02	416,38	0	24,32	0	416,38	0
Myricetin 3-O-arabinoside	$C_{20}H_{18}O_{12}$	Flavonol	450,35	25,18	NA	NA	NA	NA	25,16	0,02	450,36	-0,01
Hispidulin	$C_{16}H_{12}O_6$	Flavone	300,26	25,42	25,44	-0,02	300,26	0	25,46	-0,04	300,26	0
Peonidin	$C_{16}H_{13}O_6$	Anthocyanin	301,27	25,42	25,44	-0,02	301,27	0	25,46	-0,04	301,27	0
Kaempferide	$C_{16}H_{11}O_6$	Flavonol	299,25	25,43	25,44	-0,01	299,26	-0,01	25,48	-0,05	299,26	-0,01
Kaempferol 3-O-glucoronide	$C_{21}H_{18}O_{12}$	Flavonol	462,36	25,54	25,56	-0,02	462,36	0	25,6	-0,06	462,36	0

Luteolin 7-O-glucoronide	$C_{21}H_{18}O_{12}$	Flavone	462,36	25,54	25,56	-0,02	462,36	0	25,6	-0,06	462,36	0
Isoxanthohumol	$C_{21}H_{22}O_5$	Flavonone	354,39	25,83	25,98	-0,15	354,39	0	26,02	-0,19	354,39	0
Xanthohumol	$C_{21}H_{22}O_5$	Chalcone	354,39	25,83	25,98	-0,15	354,39	0	26,02	-0,19	354,39	0
6,8-Dihydroxykaempferol	$C_{15}H_{10}O_8$	Flavonol	318,24	26,59	NA	NA	NA	NA	26,65	-0,06	318,24	0
Myricetin	$C_{15}H_{10}O_8$	Flavonol	318,24	26,59	NA	NA	NA	NA	26,65	-0,06	318,24	0
Morin	$C_{15}H_{10}O_7$	Flavonol	302,24	27,38	27,36	0,02	302,22	0,02	27,43	-0,05	302,22	0,02
6-Prenylnaringenin	$C_{20}H_{20}O_5$	Flavonol	340,36	28,64	28,67	-0,03	340,38	-0,02	28,85	-0,21	340,38	-0,02
8-Prenylnaringenin	$C_{20}H_{20}O_5$	Flavonol	340,36	28,64	28,67	-0,03	340,38	-0,02	28,85	-0,21	340,38	-0,02
Daidzein	$C_{15}H_{10}O_4$	Isoflavonoid	254,34	29,85	29,92	-0,07	254,22	0,12	NA	NA	NA	NA

NA: Not among the most abundant in the extract.

Table S4. Flavonoids found in *MS* by RP-HPLC-MS/MS in positive mode.

				HAE				RAE				
Name	Molecular Formula	Subclass	Expected Mass (m/z)	Expected Retention Time (s)	Retention Time (s)	Retention Time Variation (s)	Observed Mass (m/z)	Mass Variation (m/z)	Retention Time (s)	Retention Time Variation (s)	Observed Mass (m/z)	Mass Variation (m/z)
Kaempferol	$C_{15}H_{10}O_6$	Flavonol	286,23	20,99	21,23	-0,24	286,24	-0,01	21,12	-0,13	286,24	-0,01
Luteolin	$C_{15}H_{10}O_6$	Flavone	286,23	20,99	21,23	-0,24	286,24	-0,01	21,12	-0,13	286,24	-0,01
Scutellarein	$C_{15}H_{10}O_6$	Flavone	286,24	20,99	21,23	-0,24	286,24	0	21,12	-0,13	286,24	0
Cyanidin	$C_{15}H_{11}O_6$	Anthocyanin	287,24	21	21,02	-0,02	287,24	0	21,24	-0,24	287,24	0
6-hydroxiluteolin	$C_{15}H_{10}O_7$	Flavone	302,23	21,13	21,18	-0,05	302,23	0	21,18	-0,05	302,23	0
Isorhamnetin	$C_{16}H_{12}O_7$	Flavonol	316,26	22,93	23,08	-0,15	316,26	0	23,09	-0,16	316,26	0
Nepetin	$C_{16}H_{12}O_7$	Flavone	316,26	22,93	23,08	-0,15	316,26	0	23,09	-0,16	316,26	0
Ramnetin	$C_{16}H_{12}O_7$	Flavonol	316,26	22,93	23,08	-0,15	316,26	0	23,09	-0,16	316,26	0
Daidzin	$C_{21}H_{20}O_9$	Isoflavonoid	416,38	24,32	24,28	0,04	416,37	0,01	24,26	0,06	416,37	0,01
Phoretin	$C_{15}H_{14}O_5$	Dihydrochalcone	274,27	24,92	25,05	-0,13	274,26	0,01	25,05	-0,13	274,26	0,01
Eriodictyol	$C_{15}H_{12}O_6$	Flavonone	288,25	24,96	25,01	-0,05	288,25	0	24,89	0,07	288,25	0
Sakuranetin	$C_{16}H_{14}O_5$	Flavonone	286,27	25,26	25,49	-0,23	286,27	0	25,41	-0,15	286,27	0
Hispidulin	$C_{16}H_{12}O_6$	Flavone	300,26	25,42	25,45	-0,03	300,26	0	25,24	0,18	300,26	0
Peonidin	$C_{16}H_{13}O_6$	Anthocyanin	301,27	25,42	25,41	0,01	301,27	0	25,41	0,01	301,27	0
Kaempferide	$C_{16}H_{11}O_6$	Flavonol	299,25	25,43	25,44	-0,01	299,26	-0,01	25,67	-0,24	299,26	-0,01
Pelargonidin 3-O-arabinoside	$C_{20}H_{19}O_9$	Anthocyanin	403,36	25,52	25,45	0,07	403,36	0	25,36	0,16	403,36	0

Kaempferol 3-O-glucuronide	$C_{21}H_{18}O_{12}$	Flavonol	462,36	25,54	25,62	-0,08	462,37	-0,01	25,29	0,25	462,37	-0,01
Luteolin 7-O-glucuronide	$C_{21}H_{18}O_{12}$	Flavone	462,36	25,54	25,62	-0,08	462,37	-0,01	25,29	0,25	462,37	-0,01
Isoxanthohumol	$C_{21}H_{22}O_5$	Flavonone	354,39	25,83	NA	NA	NA	NA	25,88	-0,05	354,41	-0,02
Xanthohumol	$C_{21}H_{22}O_5$	Chalcone	354,39	25,83	NA	NA	NA	NA	25,88	-0,05	354,41	-0,02
Myricetin	$C_{15}H_{10}O_8$	Flavonol	318,24	26,59	26,68	-0,09	318,23	0,01	26,62	-0,03	318,23	0,01
Delphidin 3-O-arabinoside	$C_{20}H_{19}O_{11}$	Anthocyanin	435,36	26,93	27,06	-0,13	435,35	0,01	26,86	0,07	435,35	0,01
Biochanin A	$C_{16}H_{12}O_5$	Isoflavonoid	284,26	27,17	27,1	0,07	284,27	-0,01	27,37	-0,2	284,27	-0,01
Geraldone	$C_{16}H_{12}O_5$	Flavone	284,26	27,17	27,1	0,07	284,27	-0,01	27,37	-0,2	284,27	-0,01
Glycitein	$C_{16}H_{12}O_5$	Isoflavonoid	284,26	27,17	27,1	0,07	284,27	-0,01	27,37	-0,2	284,27	-0,01
Methylgalangin	$C_{16}H_{12}O_5$	Flavonol	284,26	27,17	27,1	0,07	284,27	-0,01	27,37	-0,2	284,27	-0,01
Morin	$C_{15}H_{10}O_7$	Flavonol	302,24	27,38	27,39	-0,01	302,22	0,02	27,3	0,08	302,23	0,01
Isoharmnetin 3-O-glucuronide	$C_{22}H_{20}O_{13}$	Flavonol	492,39	27,7	27,71	-0,01	492,38	0,01	NA	NA	NA	NA
6''-O-acetylglycitin	$C_{24}H_{24}O_{11}$	Isoflavonoid	488,44	27,97	27,84	0,13	488,44	0	27,8	0,17	488,44	0
6''-O-malonylgenistin	$C_{24}H_{22}O_{13}$	Isoflavonoid	518,42	29,64	NA	NA	NA	NA	29,43	0,21	518,42	0

NA: Not among the most abundant in the extract.

Table S5. Stilbenes found in *MS* by RP-HPLC-MS/MS in negative mode.

					HAE				RAE			
Name	Molecular Formula	Subclass	Expected Mass (m/z)	Expected Retention Time (s)	Retention Time (s)	Retention Time Variation (s)	Observed Mass (m/z)	Mass Variation (m/z)	Retention Time (s)	Retention Time Variation (s)	Observed Mass (m/z)	Mass Variation (m/z)
Resveratrol	$C_{14}H_{12}O_3$	Stilbene	228,24	23,83	23,58	0,25	228,23	0,01	NA	NA	NA	NA

NA: Not among the most abundant in the extract.

Table S6. Stilbenes found in *MS* by RP-HPLC-MS/MS in positive mode.

					HAE				RAE			
Name	Molecular Formula	Subclass	Expected Mass (m/z)	Expected Retention Time (s)	Retention Time (s)	Retention Time Variation (s)	Observed Mass (m/z)	Mass Variation (m/z)	Retention Time (s)	Retention Time Variation (s)	Observed Mass (m/z)	Mass Variation (m/z)
Resveratrol	$C_{14}H_{12}O_3$	Stilbene	228,24	23,83	23,71	0,12	228,23	0,01	23,58	0,25	228,23	0,01
Pterostilbene	$C_{16}H_{16}O_3$	Stilbene	256,29	24,18	24,18	0	256,3	-0,01	NA	NA	NA	NA

NA: Not among the most abundant in the extract.

Table S7. Other polyphenol compounds found in *MS* by RP-HPLC-MS/MS in negative mode.

					HAE				RAE			
Name	Molecular Formula	Subclass	Expected Mass (m/z)	Expected Retention Time (s)	Retention Time (s)	Retention Time Variation (s)	Observed Mass (m/z)	Mass Variation (m/z)	Retention Time (s)	Retention Time Variation (s)	Observed Mass (m/z)	Mass Variation (m/z)
5-heneicosenylresorcinol	$C_{27}H_{46}O_2$	Alkylphenol	402,7	0,86	0,94	-0,08	401,99	0,71	NA	NA	NA	NA
Esculin	$C_{15}H_{16}O_9$	Hydroxycoumarin	340,28	26,37	NA	NA	NA	NA	26,39	-0,02	340,29	-0,01

NA: Not among the most abundant in the extract.

Table S8. Other polyphenol compounds found in *MS* by RP-HPLC-MS/MS in positive mode.

					HAE				RAE			
Name	Molecular Formula	Subclass	Expected Mass (m/z)	Expected Retention Time (s)	Retention Time (s)	Retention Time Variation (s)	Observed Mass (m/z)	Mass Variation (m/z)	Retention Time (s)	Retention Time Variation (s)	Observed Mass (m/z)	Mass Variation (m/z)
Psoralen	$C_{11}H_6O_3$	Furanocoumarin	186,16	1	NA	NA	NA	NA	0,9	0,1	186,17	-0,01
Pyrogallol	$C_6H_6O_3$	Other polyphenol	126,11	1,05	1,04	0,01	126,11	0	1,07	-0,02	126,11	0
Scopoletin	$C_{10}H_8O_4$	Hydroxycoumarin	192,16	7,98	7,86	0,12	192,16	0	8,2	-0,22	192,16	0
3,4-dihydroxyphenylglycol	$C_8H_{10}O_4$	Other polyphenol	170,16	12,87	12,67	0,2	170,17	-0,01	13,04	-0,17	170,17	-0,01
Syringaldehyde	$C_9H_{10}O_4$	Hydroxybenzaldehyde	182,17	18,69	18,83	-0,14	182,16	0,01	18,76	-0,07	182,16	0,01
Coumestrol	$C_{15}H_8O_5$	Other polyphenol	268,22	20,84	21,05	-0,21	268,23	-0,01	NE	NE	NE	NE
4-hydroxycoumarin	$C_9H_6O_3$	Hydroxycoumarin	162,44	21,13	20,97	0,16	162,14	0,3	21,36	-0,23	162,14	0,3
Protocatechuic aldehyde	$C_7H_6O_3$	Hydroxybenzaldehyde	138,12	23,56	23,47	0,09	138,13	-0,01	23,56	0	138,13	-0,01
1,4-Naphthoquinone	$C_{10}H_6O_2$	Naphthoquinone	158,15	23,83	23,8	0,03	158,15	0	23,86	-0,03	158,15	0
Juglone	$C_{10}H_6O_3$	Naphthoquinone	174,15	23,94	24,05	-0,11	174,15	0	NA	NA	NA	NA
3-methylcatechol	$C_7H_8O_2$	Alkylphenol	124,14	24,89	24,97	-0,08	124,12	0,02	24,99	-0,1	124,12	0,02
4-methylcatechol	$C_7H_8O_2$	Alkylphenol	124,14	24,89	24,97	-0,08	124,12	0,02	24,99	-0,1	124,12	0,02
Guaiacol	$C_7H_8O_2$	Methoxyphenol	124,14	24,89	24,97	-0,08	124,12	0,02	24,99	-0,1	124,12	0,02
Phlorin	$C_{12}H_{16}O_8$	Other polyphenol	288,25	24,96	25,01	-0,05	288,25	0	24,89	0,07	288,25	0
Esculetin	$C_9H_6O_4$	Hydroxycoumarin	178,14	25	25,22	-0,22	178,13	0,01	24,87	0,13	178,13	0,01

Catechol	$C_6H_6O_2$	Other polyphenol	110,11	26,13	25,96	0,17	110,1	0,01	26,15	-0,02	110,1	0,01
Esculin	$C_{15}H_{16}O_9$	Hydroxybenzaldehyde	340,28	26,37	26,43	-0,06	340,28	0	26,43	-0,06	340,28	0
Gallic aldehyde	$C_7H_6O_4$	Hhidroxibenzaldehído	154,12	35,26	35,25	0,01	154,12	0	35,3	-0,04	154,12	0
Isopimpinelin	$C_{13}H_{10}O_5$	Furanocoumarin	246,21	47,38	47,28	0,1	246,21	0	47,51	-0,13	246,21	0

NA: Not among the most abundant in the extract.
